# 2048: Two EMR query strategies to assess prevalence of adrenal incidentaloma

**DOI:** 10.1017/cts.2017.55

**Published:** 2018-05-10

**Authors:** Michio Taya, Viktoriya Paroder, Linda Haramati, Eran Bellin

## Abstract

OBJECTIVES/SPECIFIC AIMS: To compare methods of ascertaining prevalence for adrenal incidentalomas METHODS/STUDY POPULATION: Retrospective electronic medical record study using Looking Glass Clinical Analytics (Streamline Health, Atlanta, GA, USA) at an urban university medical center. All patients with CT or MR imaging of the abdomen between 1997 and 2014 were identified. Patients with a documented diagnosis (ICD-9 code or problem list) for any history of adrenal disease were excluded. The prevalence of adrenal incidentalomas was ascertained by 2 different detection strategies: (1) documented diagnosis of adrenal incidentaloma or (2) imaging reports containing in the same sentence “adrenal” and “nodule*,” “adenoma*,” or “mass*,” and not containing “no” and “adrenal” in the same sentence. Adrenal pathology surprise was further established in the second approach by excluding patients having previously undergone adrenal lab testing (cortisol, aldosterone, catecholamines, adrenocorticotropic hormone, renin) or having been registered in the cancer registry for any cancer excluding superficial skin cancers. RESULTS/ANTICIPATED RESULTS: In total, 194,624 individuals were identified in our initial search, from which 1056 were excluded for past adrenal disease (Table 1). Detection by the documented diagnosis method yielded 1578 cases (0.8%), compared with 13,697 cases (7.1%) by the imaging report method (Figure 1). Further restricting detection to true “Adrenal Surprise” by excluding those with any past adrenal lab testing and cancer history yielded 10,568 cases (6.1%). Validation studies for the 7.1% prevalence with 100 records revealed an adrenal incidentaloma positive predictive value (PPV) of 98%. When restricted to size ≥1 cm the PPV was 84%. DISCUSSION/SIGNIFICANCE OF IMPACT: Comparing our first strategy using documented diagnoses as criterion for incidentaloma as used in a recent paper by Lopez D (*Annals of Internal Medicine* 2016; 165: 533–542), we found a prevalence of 0.8% in our population similar to her 0.6%. However, when searching at the level of radiology report text, we found a prevalence ten-fold greater at 7.1%. Therefore, adrenal incidentalomas are more robustly identified by searching radiologic reports.

